# A qualitative study of stakeholders' experiences with and acceptability of a technology‐supported health coaching intervention (SHARE‐S) delivered in coordination with cancer survivorship care

**DOI:** 10.1002/cam4.7441

**Published:** 2024-07-02

**Authors:** Sarah N. Price, Thomas K. Houston, Rajani S. Sadasivam, Stacy Wentworth, Allison Chandler, Ashley Strahley, Carol Kittel, Kavitha Balakrishnan, Kathryn E. Weaver, Rebecca Dellinger, Nicole Puccinelli‐Ortega, Jinhee Kong, Sarah L. Cutrona, Kristie L. Foley, Stephanie J. Sohl

**Affiliations:** ^1^ Wake Forest University School of Medicine, Medical Center Boulevard Winston‐Salem North Carolina USA; ^2^ Atrium Health Wake Forest Baptist Comprehensive Cancer Center Winston‐Salem North Carolina USA; ^3^ UMass Chan Medical School Worcester Massachusetts USA; ^4^ Center for Healthcare Organization and Implementation Research VA Bedford Healthcare System Bedford Massachusetts USA

**Keywords:** cancer survivor, health behavior, health coaching, self‐management, survivorship care planning

## Abstract

**Purpose:**

Healthy cancer survivorship involves patients' active engagement with preventative health behaviors and follow‐up care. While clinicians and patients have typically held dual responsibility for activating these behaviors, transitioning some clinician effort to technology and health coaches may enhance guideline implementation. This paper reports on the acceptability of the Shared Healthcare Actions & Reflections Electronic systems in survivorship (SHARE‐S) program, an entirely virtual multicomponent intervention incorporating e‐referrals, remotely‐delivered health coaching, and automated text messages to enhance patient self‐management and promote healthy survivorship.

**Methods:**

SHARE‐S was evaluated in single group hybrid implementation‐effectiveness pilot study. Patients were e‐referred from the clinical team to health coaches for three health self‐management coaching calls and received text messages to enhance coaching. Semi‐structured qualitative interviews were conducted with 21 patient participants, 2 referring clinicians, and 2 health coaches to determine intervention acceptability (attitudes, appropriateness, suitability, convenience, and perceived effectiveness) and to identify important elements of the program and potential mechanisms of action to guide future implementation.

**Results:**

SHARE‐S was described as impactful and convenient. The nondirective, patient‐centered health coaching and mindfulness exercises were deemed most acceptable; text messages were less acceptable. Stakeholders suggested increased flexibility in format, frequency, timing, and length of participation, and additional tailored educational materials. Patients reported tangible health behavior changes, improved mood, and increased accountability and self‐efficacy.

**Conclusions:**

SHARE‐S is overall an acceptable and potentially effective intervention that may enhance survivors' self‐management and well‐being. Alterations to tailored content, timing, and dose should be tested to determine impact on acceptability and outcomes.

## Graphical abstract

An entirely virtual multicomponent health coaching intervention (Shared Healthcare Actions & Reflections Electronic systems in Survivorship; SHARE‐S) was evaluated through semi‐structured qualitative interviews with stakeholders (21 cancer survivor participants, 2 referring clinicians, and 2 health coaches). These interviews revealed that SHARE‐S was acceptable and impactful in producing tangible health behavior changes, improved mood, and enhanced self‐efficacy. Increased flexibility in format, frequency, timing, and length of participation and additional tailored educational materials may further enhance implementation and outcomes.

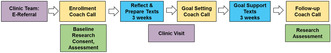

## 1 INTRODUCTION

The population of cancer survivors in the United States is large, estimated at 18 million in 2022, and expected to grow to over 22 million by 2030.^1^ Due in part to advances in early detection and treatment, the aggregate 5‐year survival rate for all cancers has risen to 67%.^2^ After completing treatment, many survivors experience complex medical and psychosocial needs, including long‐term symptoms and treatment side effects as well as risk of recurrence and second malignancies.^3^ As many of the major risk factors for these adverse outcomes are modifiable,^4^ supporting survivors' engagement in health‐protective behaviors such as completing recommended surveillance visits, quitting smoking, increasing physical activity, improving nutrition, and reducing alcohol intake is an essential component of survivorship care. In their 2005 seminal report “From Cancer Patient to Cancer Survivor: Lost in Translation”, the Institute of Medicine (IOM) highlighted the importance of assisting patients in the transition from active cancer treatment to post‐treatment follow‐up care focused on management of cancer and its late and long‐term effects.^5^

With the goal of providing patient‐centered care, enhancing communication between clinical teams, and empowering patients and their families to engage in self‐management and health‐protective behaviors, the IOM also recommended survivors be provided a survivorship care plan, a summary of treatment received and a follow‐up care plan.^5^ Given challenges with implementation and limited evidence of improved patient outcomes following survivorship care plan receipt,^6^, ^7^ clinical researchers and oncology societies have made several recommendations to optimize uptake, adoption, and impact of survivorship care planning (SCP). These recommendations include leveraging information technology and using an implementation science approach to minimize clinician burden and carefully consider the clinical context in which SCP is conducted.^8^, ^9^, ^10^ Additionally, given the time and resource constraints associated with attempting to complete the complex process of SCP in a single visit, research suggests that survivorship care plans may be more effective if delivered as part of an ongoing collaborative process focused on building patient engagement in care and facilitating adoption of self‐management behaviors (e.g., healthy lifestyles, adherence to surveillance recommendations, symptom management) rather than as one‐time information delivery.^8^, ^11^, ^12^, ^13^

The Shared Healthcare Actions & Reflections Electronic systems in Survivorship (SHARE‐S) Program is an entirely virtual multicomponent intervention designed to enhance SCP implementation and increase patient engagement before and/or after SCP visits through three main components: (1) a proactive electronic referral from the clinical survivorship care team to a health coach to connect with patients interested in SHARE‐S (e‐referral), (2) three self‐management coaching calls between patients and health coaches, and (3) automated text messages for patients designed to support the coaching process and prepare them for survivorship visits. SHARE‐S was developed based on previous self‐management interventions in the SCP context^12^, ^13^ and was designed to improve adherence to SCP guidelines that require self‐management (e.g., healthy lifestyles) through patient‐centered, preference‐sensitive goal‐setting. In a previous evaluation based on quantitative metrics (e.g., enrollment rates, patient‐reported outcome measures), SHARE‐S was found to successfully engage a patient population that was diverse in terms of race/ethnicity, age, gender, and cancer type.^14^ It was also potentially effective for improving numerous health behaviors and outcomes, including alcohol use, physical activity, fruit and vegetable intake, mindfulness, depressive symptoms, social functioning, and cancer‐specific quality of life.^14^ Although these outcomes are promising, quantitative metrics only tell part of the story, with qualitative data offering unique and valuable insights into intervention design, implementation, and impact through varied individual experiences and perspectives.^15^

As part of best practices for streamlining the pathway from effectiveness testing to routine clinical practice, qualitative feedback was obtained from key stakeholders to refine SHARE‐S and its future implementation.^16^ We conducted in‐depth qualitative interviews with patients (*n* = 21), referring clinicians (*n* = 2), and health coaches (*n* = 2) to assess the acceptability of SHARE‐S. We examined acceptability of the overall intervention and specific components based on patient participants' attitudes regarding appropriateness, suitability, convenience, and perceived effectiveness. We also considered perspectives from referring clinicians and health coaches regarding perceived effectiveness, ease of delivery, and integration with existing care structures. Understanding the acceptability of this hybrid implementation effectiveness pilot program through stakeholder perspectives may help to inform implementation not just of SHARE‐S, but also technology‐supported survivorship care interventions designed to facilitate health promoting behaviors more broadly, by identifying important elements, potential mechanisms of action, and modifications that could increase acceptability and sustainability.

## 2 METHODS

### 2.1 Clinical context and intervention

The SHARE‐S program was conducted as a collaboration between two survivorship clinics within the Atrium Health Wake Forest Baptist (AHWFB) system. Survivorship visits delivered through these two survivorship clinics were part of standard care for patients at low risk for recurrence who had completed definitive therapy. Timing of survivorship clinic referral relative to treatment completion were informed by consensus and national guidelines and determined by each cancer‐specific treatment team. Patients remaining on therapy post‐treatment (e.g., breast cancer patients on Herceptin, prostate cancer patients on androgen deprivation therapy) were offered a one‐time survivorship visit and returned to follow‐up with their primary oncologist; otherwise, patients were referred for permanent follow‐up within the survivorship clinic. Characteristics of the referring clinics have been described previously; breast, lung, prostate, and hematologic cancers were the most prevalent types treated within the clinic.^14^ Survivorship visits were conducted by advanced practice providers at one clinic and a patient navigator (registered nurse, oncology dietician) at the other. Visits focused on delivery of a survivorship care plan, addressing specific concerns, and providing appropriate surveillance and follow‐up care, including referrals to institutional and community resources for patient‐reported concerns.

**FIGURE 1:**
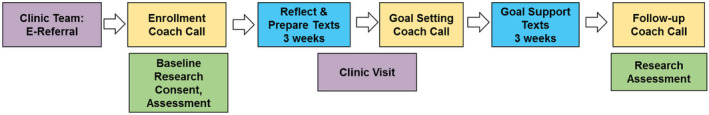
Example study flow.

Intervention development, study flow, and pilot outcomes from the SHARE‐S program are presented in greater detail elsewhere.^14^ Briefly, the intervention used a pragmatic single‐arm hybrid implementation‐effectiveness design, informed by feedback from a stakeholder advisory panel consisting of health coaches, clinicians specializing in SCP, and cancer survivors. Potential participants with upcoming or recently completed survivorship visits who were likely to meet the inclusion criteria described below were e‐referred at the discretion of the clinic scheduler or their treating clinician; approximately 29% of survivorship clinic patients were e‐referred. Prior to starting recruitment for the intervention, survivorship clinicians met with study staff to receive hands‐on demonstrations of the e‐refer tool and discuss what the patient would receive after e‐referral (see Figure 1 e.g., study flow). Clinic staff also received motivational emails during the initiation period and four proactive calls thereafter assessing barriers and strategizing solutions to successful e‐referral.

E‐referred patients were contacted by study staff, and interested participants provided informed consent through REDCap or paper mail. Participants could receive up to $100 depending on degree of study participation ($25 for each assessment, $25 for the interview). All study procedures were approved by the Wake Forest University Health Sciences Internal Review Board (IRB00064683).

Following a baseline assessment, participants received three health self‐management telephone calls (designed to be one 60‐min initial call and two 30‐min follow‐up calls) from one of three health coaches, certified by National Board for Health & Wellness Coaching‐approved training programs. One coach completed additional national requirements and certification. Two health coaches conducted most of the sessions; one served as a back‐up in the case of limited availability. Participants were provided a copy of a Personal Health Journey Guidebook, summarizing NCCN Survivorship Healthy Lifestyles Guidelines, which they were asked to review before their first coaching call. Coaching calls were designed to facilitate patient self‐management through a combination of knowledge provision (SCP overview), imagining a vision of optimal health, values reflection, self‐regulation strategies (i.e., mindfulness), goals and planning, monitoring and feedback, and social support.^14^ Coaches supported patients' autonomy by providing them a range of general topics to guide their creation of personalized health goals: (1) Eat Wisely; (2) Be Physically Active; (3) Be Tobacco Free/Limit Alcohol; (4) Strengthen Social Connections; (5) Restore (e.g., manage stress); (6) Get Adequate Rest; (7) Engage in Preventive Care; and (8) Other Personal Development (e.g., spiritual, work, finance), which were adapted from another successfully implemented telephone lifestyle coaching study.^17^, ^18^ The health coaching model adopted in this study included training in mindfulness to enhance autonomy support.^19^

Participants received automated daily text messages for 3 weeks after the first and second coaching calls (Figure 1). Texts were designed to offload clinician effort by providing information about SCP through spaced education, reminding participants to practice mindfulness or reflect on goals between sessions, and prompting reflection through brief two‐way assessments (e.g., numerical rating scale for assessing satisfaction with their current health behaviors). An example assessment would be to rate agreement with a statement such as “I am meeting my health goal” from 0 (strongly disagree) to 4 (strongly agree).

### 2.2 Participants

#### 2.2.1 Patients

Patients were eligible to participate in SHARE‐S if they were at least 18 years old, had a documented or planned cancer survivorship visit, had a working, text‐enabled phone, were cognitively able to complete study procedures (as judged by the study team), and were able to understand, read, and write English. SHARE‐S participants were eligible to participate in the post‐intervention interview after completing their last coaching session.

#### 2.2.2 Other stakeholders

Referring clinicians and health coaches were contacted to participate in interviews to share their feedback about involvement in SHARE‐S. In total, four interviews (the two primary health coaches, one referring provider from each clinic out of five total referring providers) were conducted.

### 2.3 Interviews and data analysis

#### 2.3.1 Patient interviews

Two trained researchers from the Qualitative and Patient‐Reported Outcomes (Q‐PRO) shared resource at the Wake Forest Baptist Comprehensive Cancer Center (AC and AS) conducted semi‐structured interviews with SHARE‐S participants via telephone. The semi‐structured interview guide was designed to elicit feedback on recruitment (e.g., decision to participate), programmatic components (e.g., experience with text messages, coach, goal setting), and participants' overall study experiences (feasibility, acceptability, appropriateness, perceived impact). These interviews were audio recorded and transcribed verbatim. AC and AS reviewed the transcripts and developed a codebook based on concepts found in textual data. Codes focused on programmatic components as well as emergent concepts such as accountability and personalization of the program. Data were managed with the ATLAS.ti software. All transcripts were independently coded for rigor, and the researchers met iteratively to discuss and resolve discrepancies in coding. Segments of text were reviewed by code or groups of codes and then summarized. Summaries were then synthesized into themes using the principles of reflexive thematic analysis.^20^, ^21^ Individuals who participated in the interviews were also compared to those who did not by basic demographic characteristics using t‐tests (age) and Fischer's exact test (sex, race, ethnicity).

#### 2.3.2 Other stakeholder interviews

Semi‐structured follow‐up interviews with referring clinicians and health coaches were conducted via telephone by the same Q‐PRO researchers. Interviews included questions guided by the Consolidated Framework for Implementation Research (CFIR) to assess potential barriers and facilitators in preparation for future implementation of SHARE‐S.^22^ Stakeholders were asked to provide feedback on the SHARE‐S program with a focus on the 2009 CFIR “Characteristics of Individuals” domain, including: Knowledge & Beliefs about the Intervention (e.g., effectiveness of the program, feedback on the content) and Self‐efficacy (e.g., confidence in the ability to deliver SHARE‐S, challenges, facilitators). We also asked about the CFIR “Intervention Characteristics” domain regarding Design Quality & Packaging (suggestions for workflow, additional supports that would be helpful). Following each interview, researchers reviewed the audio recording and constructed detailed field notes. Interviews were audio recorded and transcribed verbatim. Transcripts were reviewed and summarized into key points using a rapid analysis approach.^23^

## 3 RESULTS

### 3.1 Patient characteristics

Of the 40 patient participants recruited for the pilot study, 21 were invited and all agreed to participate in qualitative interviews following study completion (recruitment stopped after exceeding a number of interviews likely to reach saturation of themes^24^). Interviews were scheduled an average of 17 days after their last coaching session. Among the 21 patient participants, 17 (81.0%) were female, 6 (28.6%) identified as Black/African American or a race other than white, 3 (14.3%) lived in a rural area, and 16 (76.2%) were married or living with a partner (see Table 1 for patient characteristics). Interview participants did not differ from the rest of the sample by sex or race, and there was not enough variability in ethnicity to evaluate differences. Interviewees were younger than those who did not complete the interviews by 14.8 (SE 4.3) years (*p* = 0.002). Participants were interviewed a median of 4.5 months (interquartile range [IQR] = 1, 39.8 months) from their last treatment or surgery and most had either breast, endometrial, or prostate cancer. Interviews ranged in length from 21 to 75 (*M* = 42) min.

**TABLE 1:** Characteristics of patient participants who provided qualitative feedback for SHARE‐S (*N* = 21).

Characteristic	*N* (%)
Age (years)	
<30	5 (23.8)
30–39	0 (0.0)
40–49	3 (14.)
50–59	7 (33.3)
60–69	4 (19.0)
>70	2 (9.5)
Sex	
Male	4 (19.0)
Female	17 (81.0)
Race	
White or Caucasian	15 (71.4)
Black or African American	5 (23.8)
Other	1 (4.8)
Ethnicity	
Hispanic or Latino	1 (4.8)
Not Hispanic or Latino	20 (95.2)
Rural residence	
Yes	3 (14.3)
No	18 (85.7)
Travel time to clinic (minutes)–mean (SD)	31.6 (28.1)
Highest grade completed	
High School graduate or equivalent	2 (9.5)
Vocational or technical school/associate's degree/some college	5 (23.8)
Bachelor's degree	8 (38.1)
Graduate or professional school	6 (28.6)
Marital status	
Currently married/living with partner	16 (76.2)
Separated/Divorced	1 (4.8)
Single, never married	4 (19.0)
Difficulty in paying monthly bills	
Very difficult	2 (9.5)
Somewhat difficult	4 (19.0)
Not very difficult/not at all difficult	15 (71.4)
Number of times received medical income assistance	
Never	15 (71.4)
One to four times	4 (19.0)
More than four times	2 (9.5)
Confidence in filling out medical forms	
Extremely	17 (81.0)
Quite a bit	3 (14.3)
Somewhat	1 (4.8)
Used internet occasionally	
Yes	19 (90.5)
No	2 (9.5)
Primary tumor site	
Breast	7 (33.3)
Endometrial	3 (14.3)
Prostate	3 (14.3)
Sarcoma	2 (9.5)
Lymphoma	2 (9.5)
Brain	1 (4.8)
Ovarian	1 (4.8)
Anal	1 (4.8)
Colorectal	1 (4.7)
History of cancer‐related surgical procedure	
Yes	14 (66.7)
No	7 (33.3)
History of radiation therapy	
Yes	12 (57.1)
No	9 (42.9)
History of chemotherapy	
Yes	14 (66.7)
No	7 (33.3)
Time since last surgery/treatment (months)–median (IQR)	4.5 (1, 39.8)
Body Mass Index–mean (SD)	29.7 (6.5)

### 3.2 Patient participant perspectives

#### 3.2.1 Overall intervention

Regarding the overall intervention experience, nearly all patient participants had positive feedback and felt that the program met or exceeded their expectations (see Table 2 for themes & example quotes organized by program elements [Overall Program, Health Coaching/Health Coaches, Text Messages, and Guidebook]). Participants mentioned that the program increased their confidence and self‐efficacy in managing their health, helped them process their survivorship experience, and provided a holistic form of ongoing survivorship care. When discussing the relevance of the SHARE‐S program, some participants felt all aspects of the program were applicable, while others reported that some portions were not because they were farther out from their cancer treatment experience. Suggested changes included enrolling participants soon after finishing treatment, making the program longer, allowing participants to refer future SHARE‐S patients, more clearly explaining the specific components at the intervention outset, and tailoring intake paperwork based on time since treatment.

**TABLE 2:** Themes and patient participant quotations on specific intervention components.

Intervention aspects	Themes	Example quotes
*Overall intervention*		
Overall intervention	Enhancing self‐efficacy	“I feel like it—the concept of having a toolbox for survivorship care, which I'm not sure that I would have without the study—would be this idea that there's a lot you can gain from the study just from doing it. It might be scary to try to set these goals or figure out what you wanna put as a goal because sometimes it can be hard to figure out, but at the end of the day, I feel a lot more confident in my ability to set health‐based goals.” (P01)
	Continued support in survivorship	“…the opportunity was really good, and I think that the idea behind the process is valuable, that there is this step offered at the end because it is—I think there is the potential for people when we finish treatment—we are inundated with people taking care of us during therapy, during treatment, and there's this tendency, this potential at the end, when therapy's over, we're being seen by our physicians less, we've had our surgery, we've had everything, to feel kind of like you're floating out there on your own, with not a lot of support.” (P24)
	Holistic	“I would describe it as informative in that you learn a lot about yourself and very supportive because you're not—it's not a judgmental ‘What do you need to change?’ It's a supportive and an educational process. Also, it gives you contact from a different perspective with a treatment team. You're not just goin' in and lookin' at your blood work and lookin' at your mammograms and that type of thing. It's more holistic or part of being holistic.” (P15)
	Personal preference	“I think the intention is good, but I also think there are patients that are more receptive to that form of—I don't know what the right word is … Like I said before, I'm just not a touchy‐feely person and I just felt it was a really touchy‐feely based project.” (P18)
	Increasing number of calls	“I would love to have more phone conversations…I would have loved to communicate with her more ‘cause I really felt like I got more out of that, those sessions, than I did anything else.’” (P23)
	Program relevance	“I will say that, you know, I am so many years past having gone through treatment, and, you know, right after treatment I was seeing my doctor every three months. Then it went to six months, now it's once a year. I really feel like this program probably would have been good closer to after my treatment.” (P03)
*Specific intervention components*		
*Health coaching*		
Health coach and coaching content	Personalized	“She was great because she let me do my own setting of goals. Then we would discuss them and discuss ways that I could alleviate issues that I had identified. I felt that she really was a health coach, not a dictatorial ‘Now this is what we're gonna do. We need to talk about this.’ I thought it was helpful. I thought she did a great job as a coach.” (P15)
	Helpful in preparing for setbacks	“She asked me, ‘How are you gonna feel if you achieve it?’ and then, ‘What are you gonna do if for some reason you don't achieve your goals?’ So that was good. She was really good in preparing me for that, for setbacks, possible setbacks.”(P06)
	Accountability	“If it was short‐term goals or long‐term goals, to make it kind of one step at a time because it's like if I've never done a short‐term goal, what would be the shortest amount of time that I wanna accomplish it? If I couldn't make that goal, she would try to encourage me to try to redo it again until I can progress little by little.” (P21)
	Encouragement and positive reinforcement	“She made you feel good about the things that you have been doing and working towards and really just reassuring you that you're doing the right thing.” (P04)
	Helpful in preparation for survivorship visits	“I feel like it really helped me prepare myself to talk with my survivorship team. That was something that I was definitely nervous about because I hadn't been a part of—I hadn't been seen by a survivorship team. It was something that was a bit of a source for anxiety for me, and the coaching helped me tamp that down a little bit.” (P01)
Mindfulness exercises	Focusing and relaxing	“I actually was in awe of some of the things that came out of it. Once we started doing some of the exercises, I was surprised that things turned out the way they did, but I was glad I went through it…there was one exercise when she wanted me to just relax and relax your mind and just breathe slowly, and just don't think about nothing. I ended up being at peace, and you start thinking about, you miss your parents. It just seemed like things slowed down in that moment for me, and I wasn't really expecting that.” (P37)
	A new daily practice	“What I found really helpful were the breathing exercises. Sometimes during the conversation, she would stop, and she would get me to breathe and relax and to breathe in and out. She would ask me how I feel, and to just clear my mind or my head, get all the cobwebs out and everything. That really was helpful. Actually, I still do that every day.” (P40)
	Helpful for goal setting and achievement	“The mindfulness has helped. I pretty much try to sit down somewhere everyday and just get into my mindfulness. Especially when I get some quiet time or I'm alone. That really helps me to realize what I just went through, and help me realize the way I need to be, and what I need to do, and what I need to continue to do to try to stay healthy, and stay focused, and back to gettin' my life how it used to be.” (P35)
Coaching modality	Remote (telephone/video)	“Having the ability to do it remotely was something I really appreciated because I know not everything can be remote. It made it a lot more accessible I think, which was a huge benefit. I would definitely say I would recommend having that availability even beyond the pandemic because I know I'm more willing to complete something if I don't have to travel to do it [laughter] because I don't live super close to Wake Forest. Being able to complete all of my coaching remotely was really beneficial.” (P01)
	In‐person	“I tend to do better face to face, remotely I think it's too easy to get distracted with my husband's walking up down the hallway, or I'm seeing the pile of laundry that needs to be [laughter] done or something, so that's just me.” (P18)
*Text messages*		
Text message content	Accountability	“[The text messages] made me feel accountable. It brought back my sessions with [health coach] and bein' able to express certain things to her. It made me feel like, ‘Okay, I'm holding you accountable here of the things that you said that you wanted to do.’” (P08)
	Care	“How did I feel after I read the text messages? Well, you feel cared for. You know somebody is—somebody cares at least, trying to help you.” (P17)
	Impersonal	“I thought they were cold. Well, they didn't seem very personal, even though they're set up to be personal and say, ‘Hey, focus—think about this today,’ or ‘Think about your goal and where you are with that today.’ They didn't seem very catered specifically to me. It seemed a bit robotic.” (P23)
Text message outcomes	Facilitated reflection	“I really enjoyed the text messages. They were very specific, did not take a lot of time to respond to. What helped me was each time I would get a text message, it would force me to think about the subject, what they were asking. I also tried to use my guidebook to write down any notes that I might have or thoughts that I might have at the time and things that I wanted to discuss during our meetings. They were very important, very thought‐provoking.” (P20)
	Preparation for survivorship appointment	“I think they gave me a good segue into being able to talk about certain things with my provider. I think a lot of the questions were more so about my wellbeing that I don't really think to talk to my providers about.” (P08)
	Limited influence	“The text messages didn't really force me or make me communicate with any of my doctors. My phone conversation with [the health coach], that did prompt conversations with doctors and work and other things.” (P23)
Quality and timing of messages	Appropriate	“For me, I think it was about right. I mean, the thing with text messages, you can either act on them, or you can ignore them. I mean, it's your choice of what to do.” (P03)
	Too many or too long	“At some point it got to be a bit much. It was like, you respond and then you immediately get another one, and I really just didn't have time to just keep on and on. I think maybe the number—and this is just personally for me—the number of text messages just became burdensome at some point.” (P12)
*Personal Health Journey Guidebook*		
Outcomes of guidebook	Preparation for coaching and/or survivorship appointments	“I think the guidebook prepared me more for my appointment. I got a lot of benefit out of it just in making my own list of questions after, especially like—I don't know if we'll go through these questions, but I don't have the guidebook in front of me, but the biggest part was the wheel and grading you on different things. Then I'll develop questions to ask the doctor and things coming out of that. That really was helpful.” (P04)
	Facilitated reflection	“The wellness wheel and looking at all the things on there really helped me focus in on what I was doing at the time, and what I needed to be doing, and what was important to me. Categorizing things and putting things in order of importance, all of that was very helpful.” (P23)
	Augmented text messages	“By being able to go through my journal and scoring myself and answerin' the questions and things of that nature, it helped me really be able to look at the text messages and be like, ‘Okay, I just responded this in my journal, and so this is what it's referring to.’” (P08)

#### 3.2.2 Health coaching

All SHARE‐S participants had positive feedback about the coaches, and the vast majority had positive feedback about the health coaching component of the intervention. Participants found the coaches empathetic and genuine. They enjoyed coaches' encouragement and assistance applying behavioral techniques to help them solidify goals, change daily routines, anticipate setbacks, and prepare for survivorship visits (see Table 2). Participants described the patient‐centered, preference‐sensitive nature of the coaching intervention as empowering and enjoyed the accountability of having coaching calls. Nearly half of the participants brought up mindfulness exercises practiced in their coaching sessions and many identified mindfulness practice as the best part of the program, indicating that it benefitted their stress, mood, and sleep. Most participants reported acceptability of the coaching modality such that remote intervention delivery made their participation possible, especially participants who had to travel a long distance for care. Suggested changes related to health coaching included increasing the number of coaching calls, introducing flexibility in increasing or decreasing the length of calls, clearly communicating the video conferencing coaching option, adding options for in‐person and/or email‐based coaching, and adding group sessions to allow for perspectives of other cancer survivors.

#### 3.2.3 Text messages

Many participants found the text message content useful for keeping them on track with their goals, although a few found the content impersonal (see Table 2). Only a few participants reported text message outcomes (e.g., the texts prompted them to reflect and bring up wellbeing with their survivorship care providers); others did not find the text messages helpful in preparing for survivorship visits or found the guidebook to be more effective in this aspect. While most participants felt the quality and timing of messages received was appropriate, a few suggested making the text messages less frequent, later in the day, more personal, or more interactive.

#### 3.2.4 Guidebook

Participants were not specifically asked to provide feedback about the Personal Health Journey Guidebook provided as part of the program, but most shared spontaneous feedback, with several noting outcomes from the guidebook, including that they used it to prepare for their survivorship appointment, that it prompted reflection, and that it complemented the text messages (Table 2). Suggestions for improving the guidebook included providing more instruction for how to use it, creating an electronic and/or online version, organizing it to align with the text messages, and expanding or simplifying the wellness wheel demonstrating a healthy, balanced lifestyle.

#### 3.2.5 Health goal setting

Regardless of whether participants' initial goals were concrete (e.g., increase physical activity, improve nutrition, practice mindfulness) or more abstract (e.g., accept my diagnosis, be gentle with myself), nearly all participants reported tangible improved health behaviors and felt that the program was beneficial. The majority reported a change related to one or more health behaviors (e.g., increased physical activity, improvements in healthy or mindful eating) and reported an enhanced capacity for self‐regulation in pursuit of their goals (see Table 3 for themes and example quotes related to health goal setting and program impact). The vast majority were confident they would continue working toward their goals at the end of the SHARE‐S program. Participants described encountering external (e.g., work demands, COVID‐19 restrictions, access to walking paths for rural patients) and internal/physical (e.g., fatigue, pain) barriers in working toward their goals. However, learning to identify and plan according to these barriers was an important outcome for participants.

**TABLE 3:** Themes, sub‐themes, and patient participant quotations on health goal setting and program impact.

Themes	Sub‐themes	Example Quotes
*Health Goal Setting*		
Improved health behaviors	Nutrition and exercise	“Well, my husband I'm sure has noticed that my eating habits have changed, our eating out habits have changed. The places that we go to eat, fast food or a restaurant. I want to go somewhere that has healthy options. That has changed that I guess. And we have done some more—well since it's getting spring—we've done some more outdoor activities, some hiking, and going to the park with our dog here.” (P06)
	Tying health goals to values	“I'm not one that's like, ‘Okay, let's go work out at the gym five days a week,’ I'm more so like, ‘How can we get the exercise without feelin’ like I'm exercising and workin' out? ‘[Health coach] helped me realize that bein’ active with my son is one thing that I just love to do…now, we find ways to—even if it's just my 15‐minute lunch break while I'm at home, we'll just go outside and do something quick. If it's blowin' bubbles, if it's just goin' and sittin' in the grass and havin' a snack, we just go and get outdoors and just find ways in our day to get active.” (P08)
Enhanced capacity for self‐regulation	Anticipating and planning for setbacks	“I think I … learned the lesson that it really is important to think about barriers, that barriers are not internal. The barriers are things that I think of in my outside environment that could just be things that occur that I have no control over that would make it—as hard as I'm trying, would make it impossible for me to achieve the goal in the time that I've planned and so that identifying those is a benefit to me. They may never come up, but it does help me to plan and have maybe a plan B. I definitely learned that.” (P24)
	Learning from setbacks	“Well, they went well because when I'd meet a goal, I was pretty proud of myself. When I didn't, I would–instead of beatin' myself up, I would say, ‘Okay, this is why you didn't, and this is what you need to do.’ The goal setting was good.” (P15)
	Coping with setbacks	“I think one of the biggest perspectives that I was able to come to is that you're always gonna have potential setbacks, but your—or some days are just not gonna be your day, but every day is a new day and a new opportunity to change that.” (P04)
	Setting realistic goals	“Yeah, so in the past I've always when I wanted to work out post‐treatment I was really concerned about the stamina. One of the things that I did throughout coaching was come to the realization that it doesn't need to be this super intense workout. It can be really simple or low impact or anything, and I'll still be okay. That was a big part of my goal process.” (P01)
*Program Impact*		
Stress reduction and mood improvement	Mindfulness as stress management	“She [health coach] really helped me, gave me some breathing exercises, and it sort of calmed me because sometimes when you live alone and you don't work, your mind can be your worst enemy …” (P40)
	Enhanced ability to cope with stress	“Well, it definitely helped me focus in on the stresses and stressors in my life and at certain times and what was bothering me and focus on the specifics and address one thing at a time. What could I do right now to manage the stress that I'm feeling right now, from whatever it is? I feel like it definitely helped me to decrease my level of stress that I was experiencing, and it continues to help me.” (P23)
Improved sleep	Mindfulness enhancing sleep	“Just by those mindful moments and taking time to quiet down and clear my mind. Sleep a little better.” (P18)
	Changing sleep behaviors	“I was getting five and a half to six hours a night, because I would watch TV until I just got bored to death or really tired. Now, I'm goin' to bed. I'm not even turnin' it on. I'm getting about seven to seven and a half hours sleep a night. It makes me have a little more energy during the day.” (P22)
	Other health behavior change influencing sleep	“I was in a lotta pain and discomfort, and like I said, goin' through two or three different surgeries at one time, I wasn't sleepin' really good. I was tossin' and turnin'. I sleep pretty good right now. Pretty much pain‐free right now. I know some of that got to do with losing the weight.” (P35)
Coping with physical discomfort		“Well, with physical discomfort, there's days that are better. Some days are worse. It helps me to able to see what I can do to manage it a lot better in a way. When I do feel discomfort, I know how to deal with it.” (P21)
Improved communication		“I definitely think maybe improved communication. Communication's already a really big deal to me, but I definitely feel that I'm able to communicate my body's needs and how I'm feeling about certain instances or whatever with myself, my partner, my doctors.” (P01)

#### 3.2.6 Program impact

The majority noted improved mood and reduced stress, with several mentioning mindfulness specifically as improving their mood (stress reduction and mood improvement; see Table 3). Many participants reported that the program improved sleep, with several noting sleep benefits from changes in exercise or mindfulness practice and others noting that SHARE‐S helped them identify and eliminate a behavior that was not conducive to sleep such as staying up late watching TV. Some patients also reported that the program helped their coping with physical discomfort through health behaviors such as increased physical activity or pain improvements resulting from losing weight.

Other changes participants noticed were improved communication about their needs, peace or closure with their cancer experience, and feeling encouraged, empowered, and/or more confident. About half of participants felt that other people—typically a close family member—had noticed some of the changes, particularly changes like increased energy and physical activity, healthier eating habits, and improved sleeping habits. Suggestions for additional information, resources, or documents for goal setting included personalized resources (e.g., informational websites, links to community programs) based on their specific goals (e.g., cancer nutrition, expected side effects from cancer treatment, connecting with other survivors, specific tips for older individuals living independently); one participant suggested having an exercise coach visit their home to help them get active. Participants also suggested having a point of contact for follow‐up questions after program completion.

### 3.3 Other stakeholder perspectives

#### 3.3.1 Referring clinicians

Both referring clinicians thought SHARE‐S met patients' needs, was beneficial, and was well‐aligned with the mission of the survivorship clinic; one said that the program “supplemented” the work of survivorship clinic providers, because the clinicians are not trained in motivational interviewing and because they are limited in the amount of time they can spend with patients. Regarding suggested changes, both clinicians expressed a need for more information about the purpose, components, and end goal of the SHARE‐S program to refer potential participants more effectively. The clinicians also identified a need for patient‐facing materials on SHARE‐S that they could put in survivorship packets, on the survivorship clinic bulletin board, or in electronic announcement displays.

Suggestions for improvement for the eRefer system were to include space to share additional pertinent information beyond email and phone number (such as a patient's availability or alternate phone numbers), to have automated messages to let providers know if a patient has been previously referred, and to add SHARE‐S as a referral option in the electronic medical record template that providers use during SCP visits. One clinician felt that SHARE‐S should be introduced to patients during or immediately after the patient's first SCP visit and the other felt that the program should be introduced immediately following treatment completion. Both clinicians felt there was utility in knowing a patient's goal(s), depending on the type, with health‐related goals such as smoking cessation or weight loss being most relevant for the clinical team.

#### 3.3.2 Health coaches

Both health coaches believed the program was effective for patients based on feedback they received, noting that participants especially appreciated the mindful meditation component and the motivational and reflective aspects. A suggestion for the future was offering group sessions following the completion of individual SHARE‐S coaching sessions. Coaches' reaction to the guidebook varied; they noted most patients found it supportive although some enjoyed the personal aspects of the health coaching more than the guidebook. Both coaches believed that the text messages were generally helpful and well‐received by participants; only one coach reported receiving negative feedback about the number of messages. Suggestions for improving the training and outcomes for coaches included increasing the frequency of the study meetings and providing more training on motivational interviewing.

One coach reported that having more sessions would help enhance rapport with patients and their confidence in delivering the intervention. One coach also discussed the perceived complexity of the referral process and a lack of strong relationship between patients, coaches, and the survivorship clinic schedulers at the program outset, both of which improved over time. Both coaches had suggestions for how to make delivering SHARE‐S easier in the future, including rapport building with referring providers to increase referrals, having a flexible communication medium with patients, having more information about mindful meditation, and having cancer survivorship‐related programs available to patients. Both were interested in working with the SHARE‐S program in the future.

## 4 DISCUSSION

Qualitative feedback from patients, referring clinicians, and coaches supports the acceptability of the SHARE‐S program. The vast majority reported mostly positive experiences and attitudes toward the intervention overall as well as its specific components. Cross‐cutting themes mentioned throughout all intervention components were accountability and self‐direction, suggesting that the intervention broadly met the stated goal of being patient‐centered in enhancing self‐management behaviors (e.g., healthy lifestyles). Quantitative survey measures from the larger SHARE‐S population (*N* = 35) also indicated that SHARE‐S was perceived as feasible, acceptable, and appropriate (means for all scales >4 on a 1–5 point Likert Scale).^14^ This prior quantitative exploration also found at least a small effect (indicative of promising improvement) in the areas of mindful attention, alcohol use, physical activity, fruit and vegetable intake, days of mindfulness practice, depressive symptoms, ability to participate in social roles and activities, cancer‐specific quality of life, benefits of having cancer, and positive feelings.^14^ The present study bolsters these findings, with participants noting changes in mindfulness and mindfulness practice, physical activity, fruit and vegetable intake, reductions in stress and improvements in mood, and increased participation in valued activities. These qualitative analyses also expand quantitative findings in highlighting that patients further perceived an enhanced ability to communicate their needs with providers, especially regarding holistic concerns related to their overall well‐being. Preparing patients to broaden the scope of discussion to include lifestyle considerations and overall wellness is an important component of shifting from active cancer treatment to survivorship care.^5^

Interestingly, although interviews emphasized benefits in sleep, these changes were not clinically significant as measured by the PROMIS‐29 in quantitative analyses.^14^ Depending on patient‐identified interest and clinical appropriateness, referral to more targeted interventions may enhance patient outcomes through higher‐dose sustained participation. Behavioral changes motivated by health coaching may be most durable when supported through patient participation in evidence‐based interventions for treating insomnia, quitting smoking, managing fear of cancer recurrence, and staying active, which may be available in‐clinic, online,^25^, ^26^, ^27^ or in community settings such as the YMCA.^28^ The importance of linkage to additional resources is also reflected in participant requests for more tailored information to support their pursuit of self‐determined goals. Connecting health coaches to existing psychoeducational and other community resources (e.g., through existing databases^29^) may enhance their ability to provide nondirective support that is tailored to patients' specific needs. By improving patients' self‐determined goal setting and providing accountability through health coaching, SHARE‐S could potentially serve as a consistent touchpoint in a personalized approach to engaging patients in comprehensive self‐management.

Patient interviews also highlight potential mechanisms of action and program components that are perceived as especially important. Consistent with Self‐Determination Theory, which emphasizes the critical role of patients' autonomous engagement in making sustainable health changes,^30^, ^31^ the health coaching components were well received and linked to program effectiveness. Patients noted feeling empowered by being “in control” of their coaching sessions and appreciated the nondirective nature of the goal setting process. Additionally, patients felt confident in their abilities to sustain their health behavior changes based on their ability to set value‐driven goals that considered their broader life context (e.g., getting more activity by playing with grandchildren). These findings align with theory and empirical findings demonstrating that health behavior change goals are more likely to be achieved and maintained if they are driven by the patients themselves.^30^, ^31^, ^32^ Mindfulness exercises were identified as a favorite component and key driver of self‐determined goal achievement by both patients and coaches. Specifically, patients noted that mindfulness provided not just relaxation, but also value and goal clarification and enhanced ability to achieve goals. This aligns with previous work establishing mindfulness awareness of the discrepancies between current and desired states as a foundational motivator in behavioral change and a key promoter of self‐regulation.^30^, ^33^ Health coaching as a field recognizes that mindful presence is essential to a coaching relationship; however, not all training programs specifically incorporate mindfulness practices as tools to enhance this competency for coaches or to facilitate patients' self‐regulation.^19^ Although the qualitative nature of data collection, lack of control group, and relatively small sample size precludes direct comparison with other behavioral interventions (including those incorporating mindfulness), results indicating benefits of coaching and mindfulness are consistent with previous meta‐analyses demonstrating small‐to‐medium impacts of mindfulness‐based interventions for individuals with cancer.^34^, ^35^, ^36^ These interventions are cost‐effective, non‐pathologizing, and deliverable in a variety of formats (including eHealth/mobile health), suggesting that mindfulness training may be made accessible to and impactful for patients across the cancer continuum.

Additionally, there was some convergence in stakeholder's feedback on opportunities to improve the perceived appropriateness, suitability, and convenience of SHARE‐S. Both patients and health coaches suggested increasing program flexibility with respect to number, spacing, and length of coaching sessions and modality of coaching delivery. Specifically, both coaches and patients wanted more sessions to build rapport and enhance accountability and self‐management skills. Other health coaching interventions for cancer survivors have been longer in duration and included more sessions (e.g., 11 calls over 6 months; weekly for 6 months).^37^, ^38^ While this relatively low‐dose intervention (involving just three remotely delivered sessions with a health coach) produced promising effects, future research could expand the number of sessions, while still considering how technology may support scalability. Referring clinicians and patients reported informational needs during the referral and enrollment process; both groups noted they would benefit from more information about what to expect from the program and its specific components at its outset, suggesting that more provider training and supportive materials may be beneficial. Additionally, having the coaches provide the initial training could help strengthen relationships with clinical partners and referral resources. Clinicians and patients also suggested engaging patients sooner in their survivorship trajectory, which may be done more consistently in future research to enhance engagement and increase perceived relevance. Patients who provided this feedback (*n* = 4) were a median of 25 months since last surgery/treatment (range: 5–184 months), suggesting that it may be ideal to reach patients as soon as feasible after active treatment or potentially even earlier. Both patient and health coach interviews also suggested the use of group modality/peer mentorship as potential future directions, the feasibility of which may be explored in the future.

The results of this study must be considered within the context of its limitations. Key limitations to the present qualitative analysis are the small number of health coach and referring provider interviews, recall bias, and interview length. Although only two health coaches and two referring clinicians were interviewed, these interviewees represented 67% and 40% of each stakeholder group, respectively. Stakeholders may have had difficulty accurately recalling components of the SHARE‐S program because there was a gap in time between when they interacted with certain study components and when they completed their interviews (at most 1–2 weeks post‐intervention for patients, approximately 4 weeks for clinicians). Although participants were not asked specifically to provide feedback about the Personal Health Journey guidebook, some provided spontaneous feedback. That fact that not all participants were prompted to discuss this intervention component may impact the generalizability of results. Additionally, the mean length of the interview guide exceeded the planned 30‐min interview time. Most participants were willing to extend the discussion and the interviewers did not feel that the depth or quality of data were affected. Yet, participants may not have discussed certain questions toward the end of the interview as in‐depth as they would if they were asked within the pre‐specified time window. For future studies, piloting the interview guide could help to determine the appropriate interview length for more transparent communication with participants.

The size and composition of the patient population that participated in interviews also presents a limitation, although quantitative analyses of the entire population support findings with respect to acceptability and impact.^14^ Although quantitative analyses indicate promising reductions in hazardous alcohol use, only one participant out of 40 recruited specifically chose “be tobacco free/limit alcohol” as their goal^14^; this participant was not interviewed in the present study, limiting understanding of the acceptability and impact of SHARE‐S with respect to substance use. Participants also varied considerably in the length of time since their cancer treatment had ended, and in the complexity and duration of their treatment, which likely influenced program experiences. Although our sample size prohibits stratification by time since diagnosis or complexity of treatment, future analyses may consider how these variables moderate participant experiences and the perceived suitability and effectiveness of the intervention. An additional consideration for generalizability is the eligibility requirement of having a working, text‐enabled phone. We considered equity in the design of the study by requiring the use of cell phone numbers only (not emails) for participation in order to be more accessible to patients with limited home technology. The vast majority adults in the United States (97%) now own a cellphone of some kind.^39^, ^40^ Although the digital divide has lessened substantially over time, inequities remain. Individuals with incomes <$30,000, without a college education, and age 65+ remain least likely to own a cellphone (<95% vs. >98%).^39^, ^40^ Additionally, individuals in rural areas are more likely to have difficulties accessing high‐speed internet and black and Hispanic individuals are more likely to report having their cell phone service temporarily canceled or shut off due to financial constraints.^40^, ^41^ While none of the patients assessed for eligibility in this study were excluded for not having a working text‐enabled phone, it is possible that some patients were not referred for this reason. Finally, the majority of interviewees (61.9%) were patients with breast, prostate, or endometrial cancer. Individuals with thoracic or hematological cancers were underrepresented among both the larger pilot study population^14^ and among the subset of patients participating in interviews relative to the broader survivorship clinic population. This may represent a bias in clinician referrals or a difference in patient agreement to participate, which may limit the generalizability of results.

Nevertheless, we were able to obtain thoughtful responses from a diverse population that was representative of the larger SHARE‐S participant sample and our catchment area. Future research should aim to evaluate recruitment materials and referral strategies to further enhance participation from groups underrepresented in clinical trials and cancer research broadly.^42^, ^43^, ^44^ Given feedback that increasing flexibility in the timing and dosing of the intervention would increase its acceptability and potentially its impact, future research should explore whether these adaptations are associated with improved participation and outcomes. A future implementation trial may also elucidate the optimal amount of tailored information or timing for referral.

## 5 CONCLUSIONS

Based on feedback from clinicians and participants, SHARE‐S is acceptable; it was described as convenient, mostly appropriate/applicable, and impactful in enhancing self‐efficacy, promoting sustainable health behavior change, and improving mood, stress, and mindfulness. Future adaptations that may enhance SHARE‐S acceptability and impact include increased flexibility in intervention timing, format, and dosage, and more tailored content.

## ETHICAL APPROVAL

This trial was approved by the local Institutional Review Board and informed and performed in accordance with the ethical standards as laid down in the 1964 Declaration of Helsinki and its later amendments or comparable ethical standards.

## CONSENT TO PARTICIPATE

Consent was obtained from all individual participants included in the study.

## CLINICAL TRIAL REGISTRATION

ClinicalTrials.gov–NCT04337203

## AUTHOR CONTRIBUTIONS

**Sarah N. Price:** Conceptualization (equal); visualization (equal); writing – original draft (lead). **Thomas k. Houston:** Conceptualization (equal); data curation (equal); funding acquisition (lead); methodology (equal); resources (equal); supervision (equal); visualization (equal); writing – review and editing (equal). **Rajani S. Sadasivam:** Conceptualization (equal); funding acquisition (equal); methodology (equal); writing – review and editing (equal). **Stacy Wentworth:** Conceptualization (equal); methodology (equal); resources (equal); writing – review and editing (equal). **Allison Chandler:** Data curation (equal); formal analysis (equal); investigation (equal); visualization (equal); writing – review and editing (equal). **Ashley Strahley:** Data curation (equal); formal analysis (equal); investigation (equal); visualization (equal); writing – review and editing (equal). **Carol Kittel:** Data curation (equal); formal analysis (equal); software (equal); visualization (equal); writing – review and editing (equal). **Kavitha Balakrishnan:** Conceptualization (equal); methodology (equal); writing – review and editing (equal). **Kathryn E. Weaver:** Conceptualization (equal); methodology (equal); resources (equal); supervision (equal); writing – review and editing (equal). **Rebecca Dellinger:** Data curation (equal); project administration (equal); writing – original draft (supporting). **Nicole Puccinelli‐Ortega:** Conceptualization (equal); project administration (equal); writing – review and editing (equal). **Jinhee Kong:** Project administration (equal); writing – review and editing (equal). **Sarah L. Cutrona:** Conceptualization (equal); funding acquisition (lead); resources (equal); writing – review and editing (equal). **Kristie L. Foley:** Conceptualization (equal); funding acquisition (lead); resources (equal); writing – review and editing (equal). **Stephanie J. Sohl:** Conceptualization (equal); data curation (equal); methodology (equal); project administration (equal); resources (equal); supervision (lead); visualization (equal); writing – original draft (equal).

## FUNDING INFORMATION

This research was supported by the National Cancer Institute as a pilot study component of Grant Award Number P50CA244693 (Foley/Houston/Cutrona, Multi‐P.I.s) iDAPT (Implementation & Informatics: Developing Adaptable Processes and Technologies for Cancer Control), which is one of seven National Cancer Institute‐funded Implementation Science Centers for Cancer Control and through the Comprehensive Cancer Center of Wake Forest University Cancer Center Support Grant (P30 CA012197), which also supports the Qualitative and Patient‐Reported Outcomes Developing Shared Resource. The first author is also supported by a postdoctoral fellowship from the National Cancer Institute (T32CA122061). The content is solely the responsibility of the authors and does not necessarily represent the official views of the National Institutes of Health. In addition, the views expressed in this paper are those of the authors and do not necessarily reflect the position or policy of the Department of Veterans Affairs or the United States government.

## CONFLICT OF INTEREST STATEMENT

The authors report there are no relevant financial or non‐financial interests to disclose.

## Supporting information


Data S1:

Data S2:

## Data Availability

The data that support the findings of this study are available from the corresponding author upon reasonable request.
