# Supplementary material for: A qualitative study of stakeholders' experiences with and acceptability of a technology‐supported health coaching intervention (SHARE‐S) delivered in coordination with cancer survivorship care
Source: Cancer Med. 2024 Jul 2;13(13):e7441. doi: 10.1002/cam4.7441 (PMC11220173; doi:10.1002/cam4.7441)
Supplement: Supplementary file 1 — Data S1: [file CAM4-13-e7441-s001.docx]

**Post Intervention Patient Interview Guide**

**Introduction**

- Thank you for your time today.
- Introduce self.
- The study you experienced was a pilot phase to test our program. The purpose of our conversation today is to help our study team learn from your experience, what you found helpful and not helpful, and what you recommend that we do to improve the study.
- I consider you to be an expert consultant on your experience with the study and how we can best plan the next step. Anything you choose to share is highly valuable.
- This interview will last no longer than one hour and I will audio record our conversation so we can go back and be sure to document your feedback. If at any point, you would like to take a break, skip a question, ask a question, or stop the interview, just let me know. Do you have any questions for me before we start?

**First let’s talk about the very beginning of your involvement.**

1. When you first heard about the study what made you decide to participate?

    Who was the person who first told you about the study? What did the person say that motivated you to want to learn more about the study? What made it feel important? Did your provider recommend it?

What did you expect? In what ways did the study program meet, exceed or fail

your expectations?

1. Tell me about your experience with the person who told you more and enrolled you in the program.

In what ways did she respond to your questions and needs?

How knowledgeable was she?

**Next, let’s talk about your experience completing the SHARE-S intervention, which included texting and telephone coaching before and after your survivorship clinic visit.**

1. Tell me about your experience receiving the text messages..

In what ways did the text messages prepare you for your next coaching visit?

In what ways did the text messages affect your discussion with your health coach or your survivorship care provider?

What seemed easy to use [*feasibility*]?
What was not easy to use [*feasibility*]?

1. Overall, tell me about the content of the text messages?

In what ways was the information you received helpful or not helpful?

Were there too many messages or not enough?

What did you think about the length of the texts? *(Note. Patient Reference Sheet)*

What did you think about the response option scale? *(Note. Patient Reference Sheet – possible to shorten, such as removing “4” strongly agree)*

How did you feel after completing the activity?

1. Tell me about any problems you may have had while completing the text messages.
2. How was the study team member that helped guide you through the survivorship health coaching and goal setting activities?
    How clearly did she explain the activities?

How responsive was she to your questions and needs?

How knowledgeable was she?

How could she have made you feel even more supported?

1. What suggestions do you have for improving the SHARE-S coaching activities?

What did you think about doing coaching remotely (e.g., phone or video conference)?

**Next, let’s talk about the weeks following the SHARE-S survivorship health coaching.** You set a goal during the activity and were asked to work towards this goal.

1. How did working towards your goal go for you?

How were you successful?

What did not go as planned?

What would you do differently?

1. How important to you was the goal that you set?

How do any changes that you made fit into your life?

How much did you have to change your existing behaviors to reach your goal?
How did that work for you?

1. How likely are you to continue working towards your goal now that the study is over?
    How confident are you that you will continue to meet your goal?
2. How could we support you in working toward your goal?

What other information, discussion or resources do you need?

**Lastly, let’s talk about the program in general.**

1. Overall, what did you think about the SHARE-S survivorship health coaching program?

What did you like about it [acceptability]?
What did you not like about it [acceptability]?
What about it applied to you [appropriateness]?
What about did not apply to you [appropriateness]?

1. If you were going to describe your experience with the program to another patient, how would you describe it?
2. How do you think the program worked?
3. How did the program affect you or affect your life?

How did you feel after the program as compared to before (any different)?

Did the program change your mood or level of stress?

If so, how?

Did the program change your level of physical discomfort?

If so, how?

Did the program change your sleeping pattern?

If so, how?

Did the program change your health behaviors?

If so, how (e.g., physical activity, smoking, complementary health approaches)?

Did other people in your life notice any changes?

If so, what changes?

1. If you were directing this project, how would you make it better?
2. What else would you like to tell us?

Thank you for sharing your thoughts and experiences today!
