# Supplementary material for: A qualitative study of stakeholders' experiences with and acceptability of a technology‐supported health coaching intervention (SHARE‐S) delivered in coordination with cancer survivorship care
Source: Cancer Med. 2024 Jul 2;13(13):e7441. doi: 10.1002/cam4.7441 (PMC11220173; doi:10.1002/cam4.7441)
Supplement: Supplementary file 2 — Data S2: [file CAM4-13-e7441-s002.docx]

**Stakeholder Semi-Structured Interview Guide: Follow-up with Coaches**

**I. WELCOME**

Thank you for talking to us about your experience with the SHARE-S program. First, we would like any feedback you may have on the content of the program. We are also interested in understanding what you believe it will take to adopt this program in clinical care for cancer survivors in the future. We want to understand both the potential obstacles and the facilitators to potential future adoption of SHARE-S, so that we can plan for implementation.

Do you have any general questions for me before the interview begins?

Do you still consent to participate in this study?

Do you agree to be recorded? [Wait for participant answer] I will ask you this question again once the recording has started.

<START RECORDING>

I’ve just started the recorder. This is (interviewer name) and I am talking with participant # (participant ID). Do you consent to this audio-recorded interview?

**Introduction of SHARE-S**

We designed SHARE-S for use during the transition from active cancer treatment to survivorship, which is a particularly challenging time with an opportunity to improve upon guidance for how patients can take a more active role to optimize their health.

**II. SHARE-S Content Review**

**(CFIR Knowledge & Beliefs about the Intervention)**

1. Overall, do you think SHARE-S was effective for participants? Why or why not?
2. What comments do you have about the content used in the SHARE-S program? For example, the guidebook, coaching call guides, etc.

Follow-up questions:

1. What worked well?
2. What did not work well?
3. What might you change?
4. Anything specifically come to mind about the content of the first session? Second session? Third?
5. What comments do you have on content of the text messages?

Follow-up questions:

- 1. What messages did you find useful?
  2. What messages did you find not useful?
  3. What messages were hard to understand?
  4. What are your thoughts about the number of text messages a participant received?

Thank you for taking the time to provide feedback on the content. I am now going to ask you questions to help us plan for what it would take to implement this program in clinical care.

**Motivators and Barriers**

**(CFIR Self-efficacy)**

1. How confident were you in your ability to deliver SHARE-S?

Follow-up questions:

- 1. What gives you that level of confidence (or lack of confidence)?
  2. What would make your level of confidence higher?

1. What was challenging about delivering this program?

Follow-up questions:

- 1. What about SHARE-S seemed too complex?
  2. What suggestions do you have for making this less complex?
  3. What about it seemed awkward?
  4. What suggestions do you have for making this less awkward?

1. What would make it easier to deliver this program?
2. How do you feel about the training you received for your role?

Follow-up questions:

- 1. How were the ongoing training calls?
  2. What thoughts do you have for improving the training?

**(CFIR Design Quality & Packaging)**

1. What supports, such as online resources or a toolkit, would help you deliver SHARE-S more successfully in the future (e.g., what additional training would be helpful)?

Follow-up questions:

- 1. How would you like to access these materials?

1. What would motivate you to be involved in this program in the future?

**IV. CLOSING**

You have had a lot of really important things to say, and I appreciate your openness and willingness to share. Would you like to share any other comments before we end?

Thank you for your time.
